# Effect of prolonged pressure equalization on final drifting during pressure wire studies

**DOI:** 10.1038/s41598-024-62440-1

**Published:** 2024-05-20

**Authors:** Chien-Boon Jong, Tsui-Shan Lu, Lin Lin, Tsung-Yan Chen, Min-Tsun Liao, Jui-Cheng Kuo

**Affiliations:** 1 Department of Internal Medicine, National Taiwan University Hospital, Hsin-Chu Branch, Hsin-Chu, Taiwan; 2 College of Medicine, National Taiwan University, Taipei, Taiwan; 3 Department of Mathematics, National Taiwan Normal University, Taipei, Taiwan; 4 Department of Radiology, National Taiwan University Hospital, Hsin-Chu Branch, Hsin-Chu, Taiwan

**Keywords:** Cardiology, Medical research

## Abstract

Pressure drifting is a troublesome error in invasive coronary function tests. This study aimed to evaluate the relationship between prolonged and short-time pressure equalizations in pressure drifting. Pressure drifting was defined as the pressure gradient between the mean pressure of the distal wire sensor (Pd) and aortic pressure (Pa) when the wire was withdrawn to the tip of the guiding catheter. Significant drifts 1 and 2 were defined as the absolute values of pressure gradients > 2 and > 3 mmHg, respectively. A logistic regression model was used to evaluate the associations between prolonged pressure equalization and each pressure drifting. The prolonged pressure equalization strategy was associated with a lower incidence of drift 1 than the short-time pressure equalization strategy (6.84% vs. 16.92%, *p* < 0.05). However, no statistical differences were found in the incidence of drift 2 between the prolonged and short-time pressure equalization strategies (4.27% vs. 7.69%, *p* = 0.34). In the multivariable regression model, only the prolonged pressure equalization strategy predicted a lower incidence of pressure drift 1. In conclusion, the prolonged pressure equalization strategy was associated with a lower incidence of significant pressure drifting with more stringent thresholds than the short-time pressure equalization strategy.

## Introduction

Invasive coronary function tests are recommended for patients with angina for whom revascularization is considered to improve anginal symptoms and prognosis^1^. The use of pressure wire-based function tests has gradually increased^2^, becoming more feasible in modern catheterization rooms. However, the accuracy of studies regarding pressure-wire has rarely been reported. Pressure drifting is a common and burdensome issue associated with this test^3,4^. The drifting of the pressure wire sensor usually goes unnoticed until the pressure wire is withdrawn to the tip of the guiding catheter at the end of the procedure. A small pressure gradient between the pressure wire sensor and the guiding catheter at the aorta–coronary junction is usually clinically acceptable. However, if the gradient exceeds 2–3 mmHg, it is considered a significant drift^3,5^. In this case, repeated pressure equalization and function testing should be performed to obtain an accurate measurement.

The prevalence of significant drift ranges from 7.4 to 73% for piezoelectric sensors^4,6–9^. This large variation may be due to the differences in vendor pressure wires, the definitions of significant drifts, and operator-dependent errors. Additionally, the piezoelectric sensor’s intrinsic properties may induce a pressure drift during the test; however, the issue has been minimized by building compensation mechanisms. In contrast, a procedure-related pressure drift is an operator-dependent error and may be diminished by adhering to a standardized protocol during the procedure^5^. To date, evidence on strategies to reduce procedure-related pressure drifts remains scarce.

Pressure equalization is a pivotal step performed at the beginning of the procedure. For this procedure, the pressure wire sensor is placed at the tip of the guiding catheter, and its pressure and the pressure from the guiding catheter at the aorto–coronary junction are equalized electronically. This step makes both systems “speak the same language”^3,10^. Therefore, this study aimed to evaluate the effectiveness of prolonged vs. short-time pressure equalization on the pressure drift measured at the end of coronary function tests.

## Methods

### Study population

**Figure 1:**
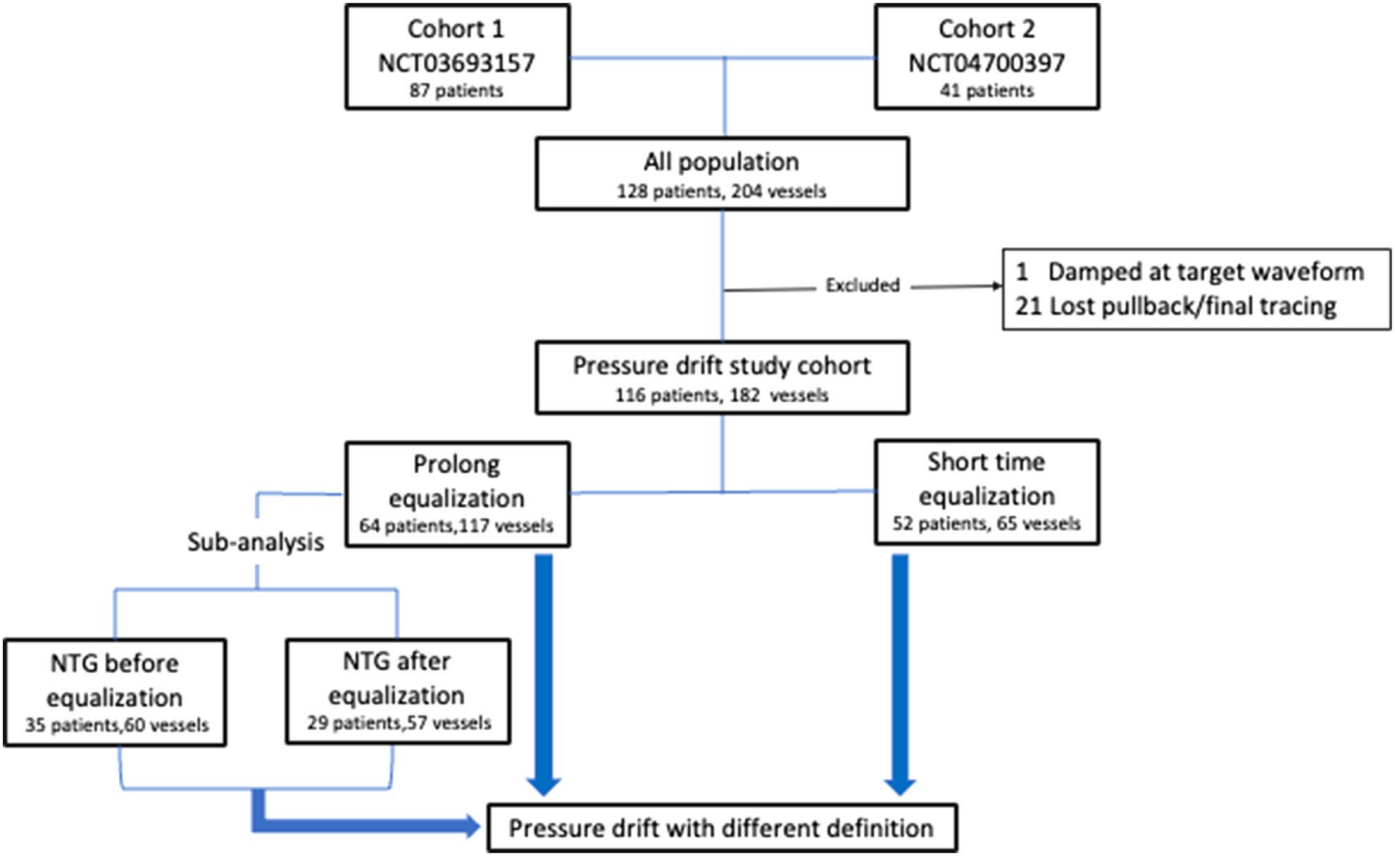
Diagram of selection of patients. The study cohort was pooled from two prospective registries. Patients with damped aortic pressure in the target waveform, those for whom no pullback/final pressure tracing was noted, or those whose data were lost were excluded from the study (22 target vessels). Overall, the final cohort comprised 116 patients and 182 vessels. The patients were categorized into the prolonged and short-time pressure equalization groups for comparison. NTG: nitroglycerin.

This observational study included the study population derived from two prospective studies (NCT03693157 and NCT04700397) conducted at our institute. The results of one of the studies (i.e., NCT03693157) have been previously published^11^. In summary, patients with intermediate stenosis (30–90% stenosis according to visual estimation) in a coronary artery requiring FFR assessment were invited to participate in these studies. Both studies had similar enrolment criteria and study protocols, except for the timing and dosage of intracoronary nitroglycerin administration, which will be discussed in the next section. This study was conducted in accordance with the principle of the Declaration of Helsinki and relevant regulations and was approved by the Institutional Review Board of the National Taiwan University Hospital Hsin-Chu Branch (IRB No. 111-155-E). All participants provided written informed consent. Patients with damped aortic pressure in the target waveform, those for whom no pullback/final pressure tracing was noted, or those whose data were lost were excluded from the study (22 target vessels). Ultimately, the final cohort comprised 116 patients and 182 vessels (Fig. 1), and the patients were categorized into the prolonged and short-time pressure equalization groups for comparison.

### Procedure and protocol of prolonged pressure equalization

First, the fluid-filled aortic pressure transducer was fixed to the table of the catheterization room at the reference height of 5 cm below the sternum^5^. The PressureWire X with a piezoelectric sensor (Abbott Cardiovascular, St. Paul, MN, USA) and COMET II with a fiber optic pressure sensor (Boston Scientific, Marlborough, MA, USA) were the two commercial pressure guidewires used in this study. Next, the wires were flushed with room-temperature saline, immersed in a fluid-filled tube for at least 1 min, and subsequently calibrated based on the manufacturers’ instructions. The pressure wire connector should remain locked unless the pressure sensor signal is interrupted. A guiding catheter without side holes was used for all procedures. The guidewire introducer needle was removed immediately after introducing the pressure wire into the guiding catheter, and the guiding catheter was flushed with normal saline before equalization. Subsequently, the Pa and Pd were equalized at the tip of the guiding catheter at the beginning of the procedure^5^. A snapshot was performed, and the pressure waveform was assessed simultaneously to prevent pressure damping or waveform distortion. Notably, the protocol of prolonged pressure equalization involved monitoring or maintaining the Pd/Pa ratio at 1.0 for at least 15 heartbeats; repeat equalization was needed if it was > 1.0 or < 1.0 during the monitoring period.

In contrast, the short-time equalization involved equalizing Pd/Pa to 1.0 once, which usually occurred quickly; it ceased immediately when the Pd/Pa ratio equalized to 1. This procedure was usually completed within five heartbeats. The number of heartbeats was measured since the actual timeframe of the equalization strategy was inconvenient to execute in practice. Intracoronary nitroglycerin at doses of 100 or 200 μg was administered before or after pressure equalization, according to the daily practice of each operator. For a long time, a short-time equalization strategy has mostly been used in our catheterization laboratory despite the recommendation of a prolonged equalization strategy by most experts^3,12^. Therefore, to minimize the risk of pressure drift, a prolonged pressure equalization strategy was suggested, although not mandated, in these registries. The equalization strategy, being non-compulsory, was registered based on the operator’s discretion. Subsequently, the coronary functional indexes were measured as previously reported^11^. Briefly, the nitroglycerine-induced acute drop of Pd/Pa was measured after introducing the distal pressure sensor to the distal third of the target vessel. Subsequently, the resting full-cycle or diastolic hyperemia-free ratio was measured after the Pd/Pa ratio became stable. Next, according to the study protocol, the doses of intracoronary adenosine administration were gradually increased from the conventional doses to higher doses during the FFR assessment. The dosage of intracoronary adenosine administration slightly differed between the two studies and the protocol of adenosine administration is shown in Supplementary Fig. S1 online. The recording of pullback or final pressure tracing was mandated at the end of the procedure.

### Data acquisition and definition of the final pressure drift

All data regarding pressure tracings were stored, and offline analysis was performed on the console of the FFR system (QUANTIEN Measurement System: Abbott Cardiovascular, St. Paul, MN, USA; POLARIS Multi-Modality Guidance System: Boston Scientific Corporation, San Jose, CA, USA) using a customized software program. A physiology team comprising one experienced cardiologist (C.B.J.) and one trained technician (R.C.K.) was considered qualified. The team evaluated the quality of the target waveform and extracted the relevant data from the pressure tracings^11^. They were blinded to the clinical data, coronary angiography results, and pressure equalization methods. The target waveform comprised the last three intact heartbeats in the pullback or final pressure tracing, which usually includes a snapshot for clarifying the drift at the end of the procedure. Moreover, the target waveform must not exhibit damping, ventricularization, waveform distortion, or signal noise. Mean pressure and heart rate were acquired at the trough of the last heartbeat’s waveform (see Supplementary Fig. S2 online). The procedure time of each functional test was calculated from the time of equalization to the end of the target waveform. Additionally, the final pressure drift was defined as the pressure gradient between the mean pressures of the Pd and Pa at the tip of the guiding catheter. A pressure gradient within ± 2 mmHg was defined as an acceptable drift^5^. Significant drift 1 and 2 were defined as an absolute pressure gradient value of > 2 and > 3 mmHg, respectively.

### Statistical analysis

Baseline characteristics were compared between the prolonged and short-time pressure equalization groups. The normality of each continuous variable was first examined using the Shapiro–Wilk test, and the Brown–Forsythe test was applied to assess the homogeneity of variances of an analyzed variable across different groups; as appropriate, the *t*-test or Wilcoxon rank sum test was subsequently used to detect the difference between the two groups. Categorical variables are presented as frequencies and were evaluated using Fisher’s exact or chi-square test. A simple logistic regression model was used to assess the effectiveness of the prolonged pressure equalization strategy for each pressure drift and no drift. The same procedure was performed in the prolonged pressure equalization group to examine the difference between the timings of intracoronary nitroglycerin administration (before vs. after pressure equalization). A multivariable logistic regression model with a stepwise algorithm was used for the analysis of drift 1, which was conditional on explanatory variables. We used the McNemar test to determine the pairwise effectiveness of any two pressure drifts and compared them with the incidence of the different definitions of pressure drift. Statistical significance was defined as *p* < 0.05, and all statistical analyses were performed using SAS version 9.4 (SAS Institute).

## Results

Tables 1 and 2 present a comparison of the demographics of the patients, vessel lesion characteristics, and medications used during the fractional flow reserve (FFR) procedure between the prolonged and short-time equalization groups. The mean age of the participants was 67 ± 10 years, and 80% of them were males. Most target vessel lesions presented 50–70% stenoses, and the median FFR was 0.81. Approximately half the target lesions had an FFR ≤ 0.80, and 54% of the target vessels were the left anterior descending artery. The median procedure time was approximately 6 min, whereas the medians of the mean aortic pressure (Pa) and heart rate at the end of the procedure were 96 mmHg and 72 beats per min, respectively.

**Table 1:** Demographics and medications used during the FFR procedure.

(Per-patient)	Prolonged pressure equalization (n = 64)	Short-time pressure equalization (n = 52)	*p* value
Age (mean)	65.3 ± 9.7	69.0 ± 11.9	0.072
Male, n (%)	52 (81.3)	41 (78.9)	0.747
Body mass index, kg/m^2^	25.5 ± 4.6	26.1 ± 4.1	0.468
Current smoker, n (%)	12 (18.8)	4 (7.7)	0.086
Hypertension, n (%)	50 (78.1)	33 (63.5)	0.082
Diabetes mellitus, n (%)	37 (57.8)	23 (44.2)	0.145
Hyperlipidemia, n (%)	50 (78.1)	41 (78.9)	0.925
Moderate to advanced chronic kidney disease, n (%)	20 (31.3)	6 (11.5)	0.011*
Atrial fibrillation, n (%)	3 (4.7)	1 (1.9)	0.417
Heart failure, n (%)	13 (20.3)	11 (21.2)	0.911
Acute myocardial infarction, n (%)	8 (13.3)	8 (15.4)	0.202
Left main disease, n (%)	6 (9.4)	2 (3.9)	0.294
Multi-vessel disease, n (%)	46 (71.9)	33 (63.5)	0.334
Left ventricular ejection fraction < 40%, n (%)	12 (18.8)	10 (19.2)	0.948
Left ventricular end-diastolic pressure, mmHg (median)	20 (13, 26)	15 (13, 20)	0.018*
Medication during the FFR procedure			
Antiplatelet, n (%)	56 (87.5)	42 (80.8)	0.319
Beta blocker, n (%)	44 (68.8)	34 (65.4)	0.701
Statin, n (%)	43 (67.2)	35 (67.3)	0.989

*FFR* fractional flow reserve.

*p < 0.05 is statistically significant.

**Table 2:** Characteristics of the target vessel and procedural profiles during the FFR procedure.

(Per-vessel)	Prolonged pressure equalization (n = 117)	Short-time pressure equalization (n = 65)	*p* value
Target vessel			
Left anterior descending artery, n (%)	63 (53.9)	36 (55.4)	0.307
Left circumflex artery or ramus intermediate artery, n (%)	30 (25.6)	11 (16.9)	
Right coronary artery, n (%)	24 (20.5)	18 (27.7)	
Lesion distribution			
Ostium to proximal part, n (%)	33 (28.2)	19 (29.2)	0.883
Tandem lesion, n (%)	36 (30.8)	10 (15.4)	0.022*
Diameter of stenosis			
30–49%	12 (10.3)	4 (6.15)	0.622
50–70%	98 (84.5)	57 (87.7)	
71–90%	6 (5.2)	4 (6.15)	
FFR value (median)	0.80 (0.72, 0.9)	0.81 (0.76, 0.88)	0.631
Duration of FFR procedure, seconds (median)	423 (346, 507)	412 (291, 558)	0.581
Aortic pressure, mmHg (mean)	94.9 ± 14.3	97.5 ± 16.6	0.274
Heart rate, beats per min (mean)	73.4 ± 13.5	71.1 ± 12.2	0.328
Guiding catheter-6 French, n (%)^a^	98 (83.8)	65 (100)	0.001*
Previous myocardial infarction at target vessel, n (%)	5 (4.3)	3 (4.6)	1.000
Maximum adenosine dose, mcg (median)	200 (200, 400)	400 (300, 800)	< 0.001*
Time to drift, seconds (median)^b^	118 (95, 145)	83 (61, 121)	< 0.001*
Final pressure drift			
Pa-Pd, mmHg (median)	0 (− 0.3, 0.6)	0.5 (0, 1)	0.002*
Pd/Pa (median)	1.00 (0.99, 1.00)	1.00 (0.99, 1.00)	0.007*

*FFR* fractional flow reserve, *Pa* the aortic pressure, *Pd* the wire-sensor pressure.

*p < 0.05 is statistically significant.

^a^Otherwise 7 or 8 French Guiding catheter was used.

^b^The time interval between the last adenosine injection and pressure record for checking drift.

**Figure 2:**
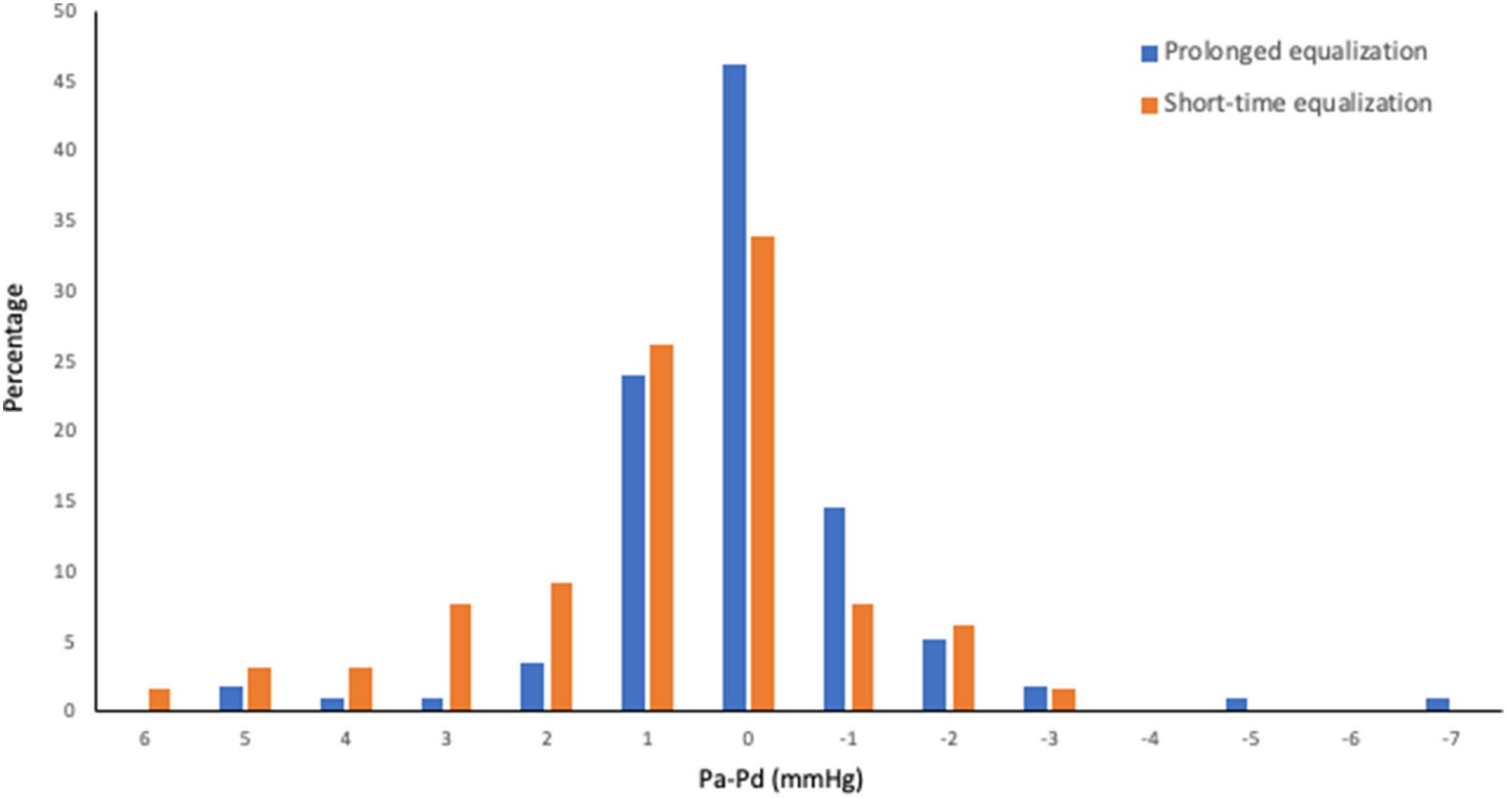
The distribution of pressure drift. The value of 0 in pressure gradient (Pa-Pd) indicates no drift. Pa: aortic pressure; Pd: wire-sensor pressure.

Figure 2 and Supplementary Fig. 3 online shows the distribution of pressure drift between the two groups (prolonged vs. short-time pressure equalization groups). The drift values were smaller, and the tails of the distribution shrunk with prolonged equalization. (*p* = 0.032, using the Brown–Forsythe test, indicating the inequality of the two variances).

Approximately half the procedures ended without pressure drifting when prolonged equalization was performed, whereas drifting was averted in one-third of the procedures when only short-time pressure equalization was performed. The prolonged equalization strategy was associated with a lower incidence of drifts 1 than short-time equalization (6.84% vs. 16.92%, *p* = 0.039, Table 3). However, no significant differences were found regarding the incidence of drift 2 between the prolonged and short-time equalization groups (4.27% vs. 7.69%, *p* = 0.34).

**Table 3:** Odds ratios of different equalization strategies on pressure drifting during pressure wire studies.

	Prolonged pressure equalization Event number (%)	Short-time pressure equalization Event number (%)	Crude OR (95% CI)	*p* value
No drift^a^	54 (46.15%)	22 (33.85%)	1.68 (0.89–3.14)	0.108
Drift 1^b^	8 (6.84%)	11 (16.92%)	0.36 (0.14–0.95)	0.039*
Drift 2^c^	5 (4.27%)	5 (7.69%)	0.54 (0.15–1.92)	0.339

*CI* confidence interval, *OR* odds ratio.

*p < 0.05 is statistically significant.

^a^Value measured with a mean pressure gradient = 0.

^b^Value measured with an absolute mean pressure gradient value > 2 mmHg.

^c^Value measured with an absolute mean pressure gradient value > 3 mmHg.

In the sub-analysis of the prolonged equalization strategy, no difference was found between the timings of intracoronary nitroglycerin administration (before vs. after pressure equalization) on the final pressure drifting (*p* > 0.05 in all drifts) (Table 4).

**Table 4:** Comparison of the timings of intracoronary nitroglycerin administration, stratified by before vs. after the prolonged pressure equalization strategy.

	Before equalization (n = 60) Event number (%)	After equalization (n = 57) Event number (%)	Crude OR (95% CI)	*p* value
No drift^a^	28 (46.67%)	26 (45.61%)	0.96 (0.46–1.98)	0.909
Drift 1^b^	5 (8.33%)	3 (5.26%)	0.61 (0.14–2.68)	0.514
Drift 2^c^	3 (5%)	2 (3.51%)	0.69 (0.11–4.30)	0.692

*CI* confidence interval, *OR* odds ratio.

*p < 0.05 is statistically significant.

^a^Value measured with a mean pressure gradient = 0.

^b^Value measured with an absolute mean pressure gradient value > 2 mmHg.

^c^Value measured with an absolute mean pressure gradient value > 3 mmHg.

In univariate and multivariate analyses, the prolonged pressure equalization strategy predicted a lower incidence of pressure drift 1 than the short-time pressure equalization strategy (odds ratio: 0.36, 95% confidence interval: 0.14–0.95, *p* = 0.04, Table 5).

**Table 5:** Prediction factors of drift 1 in pressure wire studies.

	Crude OR (95% CI)	*p* value
Prolonged equalization	0.36 (0.14–0.95)	0.039*
Duration of FFR procedure^a^	1.00 (0.93–1.08)	0.949
Lower aortic pressure^b^	0.85 (0.33–2.19)	0.731
Lower heart rate^c^	0.93 (0.36–2.42)	0.888
Age > 65 years	1.32 (0.50–3.53)	0.577
Hypertension	0.85 (0.29–2.52)	0.771
Moderate-to-advanced chronic kidney disease	0.40 (0.09–1.81)	0.235
Presentation of acute myocardial infarction	1.35 (0.37–4.92)	0.646
Left main disease	1.61 (0.43–6.10)	0.483
Lower left ventricular end-diastolic pressure^d^	1.78 (0.67–4.75)	0.250
Tandem lesion	1.06 (0.36–3.13)	0.912
Maximum adenosine dose	1.00 (1.00–1.00)	0.654
Time to drift^e^	1.00 (1.00–1.01)	0.188

*CI* confidence interval, *FFR* fractional flow reserve, *OR* odds ratio.

*p < 0.05 is statistically significant.

^a^30 s as per unit.

^b^Aortic pressure lower than the median value, 96 mmHg.

^c^Heart rate lower than the median value, 72 beats per min.

^d^Left ventricular end-diastolic pressure lower than the median value, 18 mmHg.

^e^Time interval between the last adenosine injection and pressure record for checking drift, measured in s.

Overall, the differences in the incidence rate between any drift and drift 1, any drift and drift 2, and drift 1 and drift 2 were all significant (*p* < 0.05, see Supplementary Fig. S4 online).

## Discussion

The pressure drift at the end of wire-based function tests is a non-negligible issue in physiology-guided coronary intervention because of its high incidence^4,7,9^, and the treatment strategy may require reclassification after repeated measurements^5^. To our knowledge, this is the first study to demonstrate that prolonging pressure equalization at the initiation of an invasive coronary function test may lower the incidence of significant pressure drift at the end of the procedure. Additionally, the timing of intracoronary nitroglycerin administration (before or after pressure equalization) did not significantly influence the occurrence of pressure drifts. However, our results show that the incidence of pressure drift may differ when different definitions of pressure shift are used.

Procedure-related pressure drift is an avoidable error, and several causes, mechanisms, and management techniques for pressure drift have been suggested^5^. Optimization of the pressure equalization procedure has been emphasized in only a few clinical trials, although a high incidence of pressure drifts has been noticed in many multicenter registries^4,7^. Pressure equalization for 10 s was recommended in the Functional Lesion Assessment of Intermediate Stenosis to Guide Revascularization (DEFINE-FLAIR) trial^12^. Furthermore, Pijls and De Bruyne suggested prolonging equalization by 20–30 s^3^.

However, none of the abovementioned studies showed the effectiveness of prolonged pressure equalization. The pressure equalization time was usually short and ceased immediately when the wire-sensor pressure (Pd)/Pa ratio equalized to 1 due to the rush hour in daily catheterization laboratories. Our study compared the effect of short-time equalization, which is usually performed within 5 heartbeats, with prolonged equalization, which intentionally proceeds for 15 heartbeats. The results showed a lower risk of significant drift in the prolonged equalization strategy than in the short-time equalization strategy, which may result in 6-min time savings when repeated FFR measurements are waived. Additionally, the risk of repeated wiring can be prevented, which is particularly important in high-risk procedures, such as zero-contrast percutaneous coronary interventions in patients with advanced chronic kidney disease.

We also examined several risk factors that possibly influence the incidence rate of pressure drift. First, the time for the FFR measurement, which correlated with thermal drift, did not lead to significant drift. Second, neither the maximum adenosine dose nor the time interval between the last adenosine injection and pressure record for checking drift (Time to drift) predicted the incidence of pressure drift. The distribution of the time interval between the last adenosine administration and drift check has been presented on Supplementary Fig. S4 online. Third, the other risk factors mentioned in previous studies, such as high blood pressure, old age, and left main stenotic lesion, were also not associated with significant drift^13^.

The entrapment of air microbubbles in the cavity of the wire sensor and electrical-thermal instability causes true drifts. However, the prolonged equalization strategy may allow the air microbubbles to be removed from the cavity of the wire sensor^3^. Additionally, the damped or interrupted pressure waveform is easily recognized during a prolonged monitoring period. Repeated equalization is required after amending the damped waveform. Moreover, the resting Pd/Pa is calculated by averaging the mean pressure of 3–5 heartbeats^5^, and prolonging the equalization time can minimize measurement bias error, particularly in situations of dramatically fluctuating blood pressure. These advantages of prolonging the equalization time may contribute to a reduction in pressure drift at the end of the procedure.

The clinically acceptable threshold of pressure drift differs, with the range between ± 2 and ± 3 mmHg being most commonly used^4–6,12,13^. A wider threshold (± 3 mmHg) causes fewer data to be excluded with a higher risk of treatment strategy reclassification, whereas the reverse occurs when a more stringent threshold (± 2 mmHg) is employed. In our study, the incidence rate of the more stringent thresholds was statistically higher than that of the wider thresholds. However, Cook et al. reported in a cohort of patients with true intermediate stenosis that in the more stringent threshold range, 21 and 25% of FFR and instantaneous wave-free ratio measurements would cross over at the binary ischemic cut-offs, respectively^5^. These crossover rates will be higher when a wider threshold is used than when a more stringent threshold is employed. However, the impact of such thresholds on the crossover rate depends on the values’ closeness to the binary cut-off^3,5^: the highest and lowest crossover rates occur in values nearest to and farther from the binary cut-offs, respectively. This is a mathematical phenomenon; therefore, every effort to minimize drift values should be encouraged, and a more stringent threshold is favored in vessels with true intermediate stenosis. In this study, drift 2 had a lower event rate than drift 1, and the event rate in the prolonged equalization strategy was not statistically lower than that in the short-time equalization strategy regarding pressure drift with a wider threshold. The small sample size and a lower incidence rate may have caused this statistical insignificance.

Conversely, if the final pressure drift were defined as the ratio of Pd/Pa, this would mean that none of the drifts had a Pd/Pa equal to 1.00, and significant drift was subsequently defined as Pd/Pa ratio exceeding 1.00 ± 0.02 (drift 3) and ± 0.03 (drift 4). The results were similar to the pressure gradient threshold because the prolongation of the pressure equalization strategy was associated with a lower risk of significant drift when defined by a stricter threshold of pressure ratio (Pd/Pa) (see Supplementary Tables S1 and S2 online). However, a lower incidence of any pressure drift was noted when defined by the pressure ratio (Pd/Pa) than by the pressure gradient (Pa-Pd) (52.75% vs. 58.24%, *p* = 0.04, see Supplementary Fig. S5 online). No significant differences were found in the incidence rates between drift 1 and drift 3 and between drift 2 and drift 4 (*p* > 0.05), whereas the incidence rate differences between drift 1 and drift 4 and between drift 2 and drift 3 were all significant (*p* < 0.05, see Supplementary Fig. S5 online). These results demonstrate that different definitions of significant drift might lead to a large variation in the reported pressure drifting rates in recent literature. Although the incidence of pressure drift in pressure wire procedure was high, only a few reports in clinical registries exist, and data in all-comer, real-world analysis are inadequate. Therefore, a practical, logical, and consistent definition of significant drift is required to investigate the true incidence rate of pressure drift among different institutes.

This study had some limitations. First, the nature of the observational registry only demonstrates the association between prolonged pressure equalization and significant drift rather than their causal relationship. However, our results support the recommendation that experts have provided for years^3,12^. Second, the smaller size of the guiding catheter may have affected the pressure waveform and equalization procedure. Ninety percent of the guiding catheters used were 6 French (F); otherwise 7F or 8F guiding catheter was used. No significant drift was found in vessels with 7F or 8F guiding catheter. Although not significantly different according to the univariate analysis, the effect of the 6F guiding catheter on significant drift remained elusive (*p* > 0.05, data not shown). Third, the fiber-optic pressure sensor wire, which logically may reduce the pressure drift, was used in the three target vessels included in the prolonged pressure equalization group, although none of the three procedures showed any significant drift at the end of the procedure. Contrarily, none of the fiber-optic pressure sensors were used in the short-time pressure equalization group; therefore, the assumption of the logistic regression model would be violated. We assumed that the specific FFR device could not be incorporated into the univariate analysis and that the relatively lower proportion of fiber-optic sensors would not affect this study’s results. Fourth, the prolonged pressure equalization strategy was the sole predictor of significant drift in both univariate and multivariate analyses. Specifically, the prolonged pressure equalization strategy remained once retained in the final model after processing the stepwise algorithm, although all variables in Table 5 were included in the regression model. Therefore, a selection bias and other residual confounding factors may exist. Lastly, the limited sample size and single-center experience may limit the generalization of the results to other catheterization laboratories. Therefore, further international, multicenter, randomized clinical trials are warranted in the future.

In conclusion, prolonging the pressure equalization time when initiating a pressure wire-based procedure was associated with a lower risk of significant pressure drift at the end of the procedure. Neither the procedure time of FFR measurement nor the timing of nitroglycerin administration was associated with significant pressure drift. Furthermore, this is the first evidence of the benefits of lowering pressure drift during wire-based coronary functional tests.

### Supplementary Information

Supplementary Information.

## Data Availability

The original contributions presented in the study are included in the article/supplementary material; further inquiries can be directed to the corresponding author (C-BJ).
