# Supplementary material for: Effect of prolonged pressure equalization on final drifting during pressure wire studies
Source: Sci Rep. 2024 May 20;14:11504. doi: 10.1038/s41598-024-62440-1 (PMC11106059; doi:10.1038/s41598-024-62440-1)
Supplement: Supplementary file 1 — Supplementary Information. [file 41598_2024_62440_MOESM1_ESM.docx]

Effect of prolonged pressure equalization on final drifting during pressure wire studies

Chien-Boon Jong, MD^1,2^*; Tsui-Shan Lu^3^; Lin Lin^1^; Tsung-Yan Chen^1,2^; Min-Tsun Liao^1,2^; Jui-Cheng Kuo^4^

^1^ Department of Internal Medicine, National Taiwan University Hospital, Hsin-Chu Branch, Hsin-Chu, Taiwan;

^2^ College of Medicine, National Taiwan University, Taipei, Taiwan;

^3^ Department of Mathematics, National Taiwan Normal University, Taipei, Taiwan;

^4^ Department of Radiology, National Taiwan University Hospital, Hsin-Chu Branch, Hsin-Chu, Taiwan;

***** Correspondence: jgboon0407@gmail.com; Tel.: +886-3-5326151 ext. 2010

**Supplementary Materials**

**
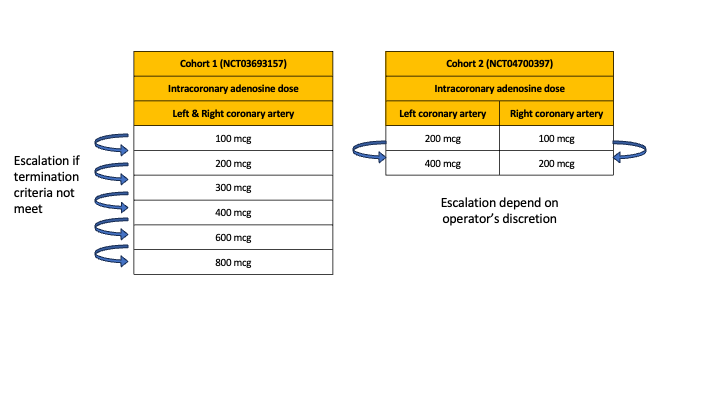
**

**Supplementary Figure S1**. The escalation protocol of the intracoronary adenosine administration during FFR assessment in the two prospective studies.

The escalation strategy of adenosine administration slightly differed between the two cohorts.


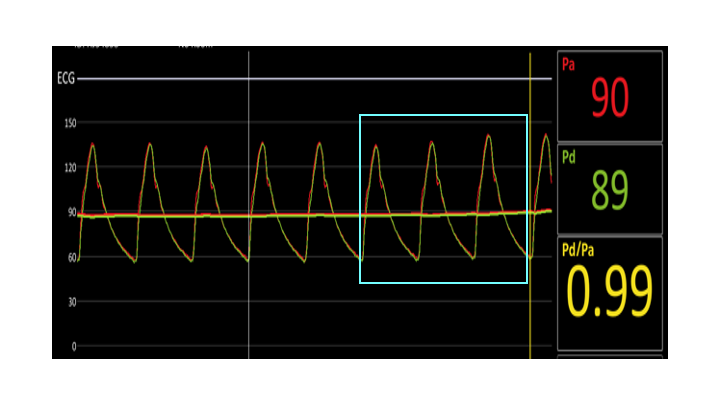


**Supplementary Figure S2**. Acquisition of the target waveform and mean pressure during pressure drift assessment.

The target waveform is the last three aortic pressure waveforms in the pullback or final pressure tracing, as shown in the light blue box. The mean pressure of the aorta (red) and wire sensor (green) acquired at the trough of the waveform of the last aortic pressure tracing (yellow line).

**
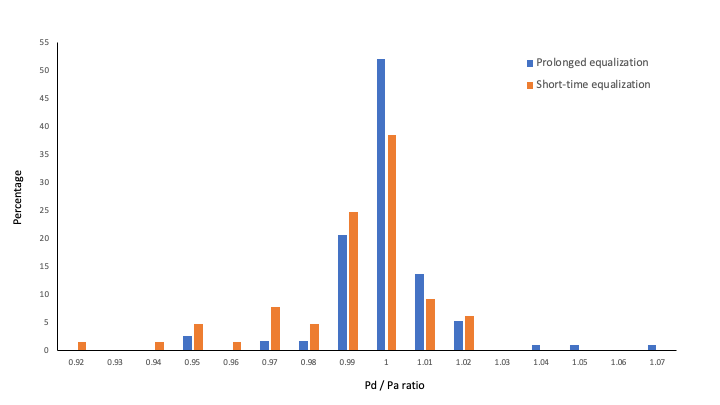
**

**Supplementary Figure S3.** The distribution of pressure drift.

The value of 1 in Pd/Pa ratio indicates no drift.

Pa: aortic pressure; Pd: wire-sensor pressure

**
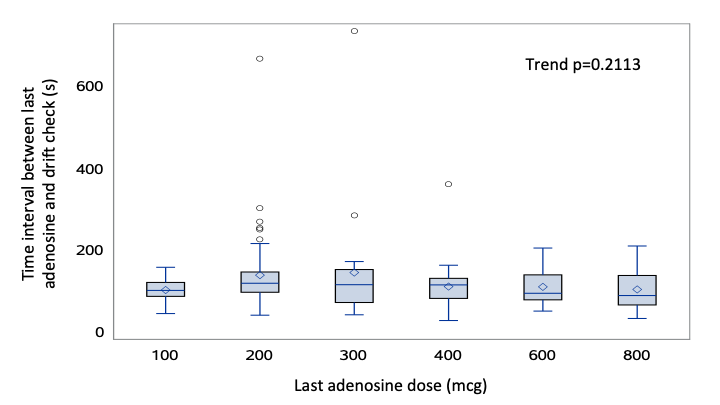
**

**Supplementary Figure S4.** The distribution of the time interval between the last adenosine administration and drift check.

The time interval between the last adenosine injection and pressure record for checking drift was not significantly different between the different administration doses of adenosine. (Trend p=0.2113)


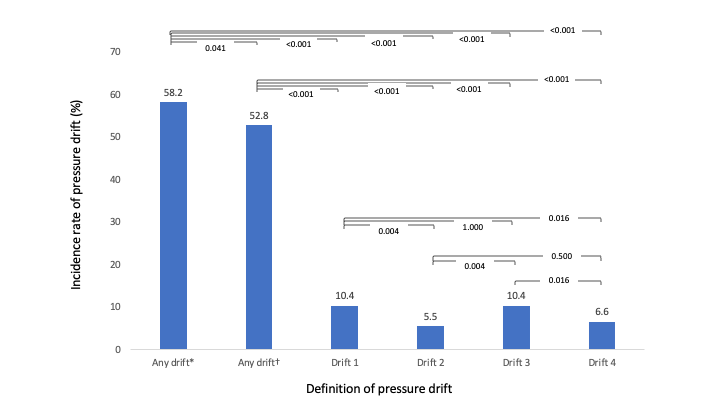


**Supplementary Figure S5**. **Comparison of the different definitions of pressure drifting in the entire study cohort**
The incidence rates of pressure drifting were significantly different among the different definitions, except for drift 1 vs. drift 3 and drift 2 vs. drift 4.

^*^Value measured with the mean pressure gradient

^*^Any drift measured with the mean pressure gradient, mean Pa-Pd not equal to 0.

^†^Any drift measured with the mean pressure ratio, mean Pd/Pa not equal to 1.

Drift 1 was defined as an absolute mean pressure gradient (Pa-Pd) value >2 mmHg.

Drift 2 was defined as an absolute mean pressure gradient (Pa-Pd) value >3 mmHg.

Drift 3 was defined as a mean pressure ratio (Pd/Pa) exceeding the range of 1.00±0.02.

Drift 4 was defined as a mean pressure ratio (Pd/Pa) exceeding the range of 1.00±0.03.

**Supplementary Table S1.** Odds ratio of the different equalization strategies on pressure drifting during pressure wire studies

|  | Prolonged pressure equalization  Event number (%) | Short-time pressure equalization  Event number (%) | Crude OR  (95% CI) | p-value |
| --- | --- | --- | --- | --- |
| No drift^a^ | 61 (52.14%) | 25 (38.46%) | 1.74 (0.94–3.23) | 0.078 |
| Drift 3^b^ | 8 (6.84%) | 11 (16.92%) | 0.36 (0.14–0.95) | 0.039* |
| Drift 4^c^ | 6 (5.13%) | 6 (9.23%) | 0.53 (0.16–1.72) | 0.292 |

*p<0.05 is statistically significant

^a^Value measured with mean pressure ratio=1.00

^b^Value measured with mean pressure ratio exceeding the range of 1.00±0.02

cValue measured with mean pressure ratio exceeding the range of 1.00±0.03

CI: confidence interval; OR: odds ratio.

**Supplementary Table S2**. Comparison of the timings of the intracoronary nitroglycerin administration, stratified by before vs. after the prolonged pressure equalization strategy

|  | Before equalization  (n=60)  Event number (%) | After equalization  (n=57)  Event number (%) | Crude OR  (95% CI) | p-value |
| --- | --- | --- | --- | --- |
| No drift^a^ | 28 (46.67%) | 33 (57.89%) | 1.57 (0.76–3.26) | 0.225 |
| Drift 3^b^ | 5 (8.33%) | 3 (5.26%) | 0.61 (0.14–2.68) | 0.514 |
| Drift 4^c^ | 4 (6.67%) | 2 (3.51%) | 0.51 (0.09–2.89) | 0.446 |

^a^Value measured with mean pressure ratio=1.00

^b^Value measured with mean pressure ratio exceeding the range of 1.00±0.02

cValue measured with mean pressure ratio exceeding the range of 1.00±0.03

CI: confidence interval; OR: odds ratio.
